# A review of metallic nanoparticles: present issues and prospects focused on the preparation methods, characterization techniques, and their theranostic applications

**DOI:** 10.3389/fchem.2024.1398979

**Published:** 2024-08-14

**Authors:** Mona Shahalaei, Abul Kalam Azad, Wan Mohd Azizi Wan Sulaiman, Atefeh Derakhshani, Elmira Banaee Mofakham, Mireia Mallandrich, Vinoth Kumarasamy, Vetriselvan Subramaniyan

**Affiliations:** ^1^ Biomaterial Group, Nanotechnology and Advanced Materials Department, Materials and Energy Research Center, Karaj, Iran; ^2^ Department of Pharmaceutical Technology, Faculty of Pharmacy, University College of MAIWP International (UCMI), Kuala Lumpur, Malaysia; ^3^ Department of Tissue Engineering and Applied Cell Sciences, Faculty of Advanced Technologies in Medicine, Tehran University of Medical Sciences, Tehran, Iran; ^4^ Department of Pharmacy, Pharmaceutical Technology and Physical-Chemistry, Faculty of Pharmacy and Food Sciences, University of Barcelona, Barcelona, Spain; ^5^ Department of Parasitology and Medical Entomology, Faculty of Medicine, Universiti Kebangsaan Malaysia, Kuala Lumpur, Malaysia; ^6^ Department of Medical Sciences, School of Medical and Life Sciences, Sunway University, Sunway, Malaysia

**Keywords:** metallic nanoparticles, theranostics, targeted delivery, genetic manipulations, gene therapy

## Abstract

Metallic nanoparticles (MNPs) have garnered significant attention due to their ability to improve the therapeutic index of medications by reducing multidrug resistance and effectively delivering therapeutic agents through active targeting. In addition to drug delivery, MNPs have several medical applications, including *in vitro* and *in vivo* diagnostics, and they improve the biocompatibility of materials and nutraceuticals. MNPs have several advantages in drug delivery systems and genetic manipulation, such as improved stability and half-life in circulation, passive or active targeting into the desired target selective tissue, and gene manipulation by delivering genetic materials. The main goal of this review is to provide current information on the present issues and prospects of MNPs in drug and gene delivery systems. The current study focused on MNP preparation methods and their characterization by different techniques, their applications to targeted delivery, non-viral vectors in genetic manipulation, and challenges in clinical trial translation.

## 1 Introduction

Green nanotechnology, or nanotechnology, is an important science that deals with the development of particulate systems with a nanosize range of 1 nm–100 nm to encapsulate living cells to deliver through biological pathways (Vijayaram et al., 2024). Particles in the nanosize range have shown improved physio-chemical properties in terms of morphology and biodistribution, which are not exhibited by larger particles of bulk material (Willems, 2005). Due to their high superficial volume ratio, the potential to interact with the molecular or cellular process, and the possibility of affecting their functions, nanoparticles are widely attractive for various biomedical applications, organic and inorganic chemistry, molecular biology, and physics (Nath and Banerjee, 2013). Metallic nanoparticles, or metal nanoparticles (MNPs), are a newly emerged form of nanoparticles (Rai et al., 2017; Ali et al., 2021). MNPs have gained significant recognition in many research areas, as they have been suggested as a promising alternative tool for targeted-site, sustained, and controlled drug delivery attributed to their novel size-dependent behavior and related properties (Sengani et al., 2017). Furthermore, MNPs may be produced using a straightforward laboratory technique in a variety of size ranges with a low dispersity index. They are also biocompatible, non-toxic, and inert. Because of their straightforward structure and ease of synthesis, MNPs offer straightforward and flexible surface functionalization. Because of these characteristics, MNPs offer a promising platform for the binding of targeting ligands on the surface at low core diameter sizes, making them ideal for use in drug delivery systems (Sathali et al., 2012).

MNPs have various reaction response mechanisms and can easily penetrate the target organs and cross biological membrane barriers. MNPs can alter cellular function by binding with cellular proteins and nucleic acids and expressing enhanced biological activity due to their smaller particle size and correspondingly high surface area (Nasrollahzadeh et al., 2015). Gold and silver have obtained more attention than other MNPs (Punjabi et al., 2015). For instance, gold is widely used in medicines, and its nanoparticles are used in different drug delivery and diagnostic cases (Bhumkar et al., 2007; Narayanan and Sakthivel, 2008; Jafari et al., 2018). The wide application and recognition of MNPs as targeted drug delivery vehicles are mainly attributed to their high degree of drug-carrying capacity with the least toxicity (Das et al., 2024) and fewer side effects despite the high loading of drugs with various coatings like polymers, SiO_2_, inorganic metals, *etc.* They are used to modify the surface of MNPs to make them appropriate for drug delivery.

### 1.1 Methods of synthesizing MNPs

**FIGURE 1:**
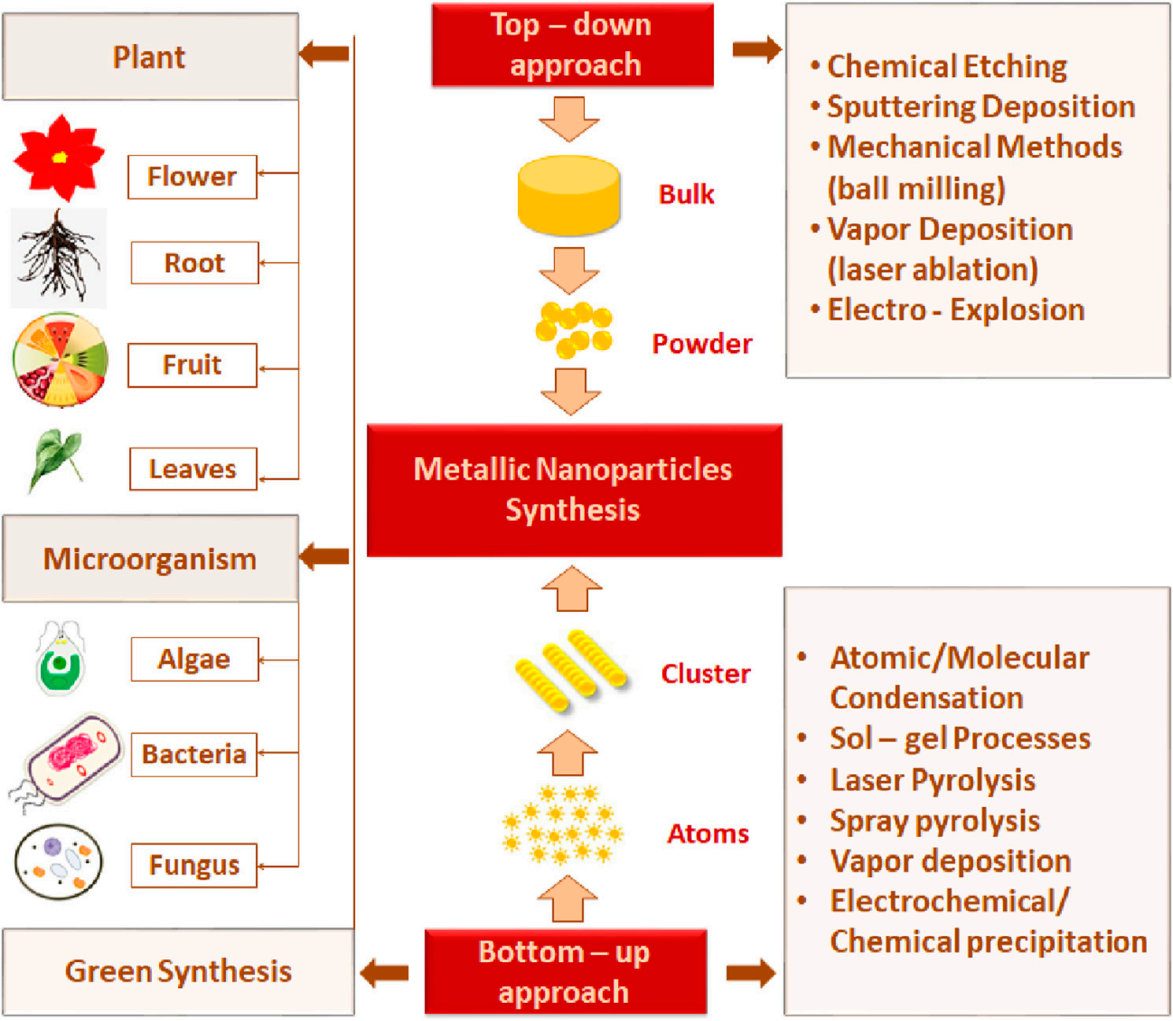
Schematic illustration of MNP synthesis methods.

The two most commonly investigated approaches to acquiring nanomaterials of desired features are the top-down method (dispersion) and bottom-up (condensation) method (Figure 1).

### 1.2 Top-down method

The top-down method is a destructive approach that utilizes the concept of decreasing the size of starting bulk metals l mm to a nanosized range using various physical and chemical treatments (Isaacoff and Brown, 2017). This approach includes mechanical methods (ball milling), sputtering deposition, and vapor methods. In the ball-milling method, bulk powder is subjected to high-energy hitting impact from the rotating solid balls in a series of parallel layers. The balls may roll down on the surface of the chamber containing bulk material (Yadav et al., 2012; Rajput, 2015).

The mechanochemical method combines the mechanical and chemical characteristics of a metal at its molecular level, and nanoparticles can be obtained by applying the mechanical aspects of a ball mill operating at low temperatures and with the use of a size-reducing agent to perform the chemical reaction (Paskevicius et al., 2009; Ghorbani, 2014). Vapor deposition requires high temperature or pulse irradiation and contains laser ablation and gas evaporation. Laser ablation depends on laser irradiation. The bulk target material is exposed to a pulsed laser and heated to its boiling point. This leads to the fragmentation of the target, which condenses to produce MNPs (Ghorbani, 2014). Bimetallic nanoparticles (BMNPs) such as Mf-AI and ZnoAl are produced. MNPs can be synthesized by the combination of two metals (bimetallic), three metals (trimetallic), or more than three metals. Magnesium nanoparticles are preferred over monometallic particles due to their satisfactory stability, catalytic, and selectivity activity. They are widely used in various fields, with a particular focus on protein conjugation and modified drug delivery (Pankhurst et al., 2003). Sharma et al. (2019) reported that the metal changed its state from its molten state into a chamber charged with inert gas following the gas evaporation technique. MNPs are formed by the condensation of the gaseous metals. The purity and type of the inert gas atmosphere influence the properties of the produced nanoparticles (Swihart, 2003; Ghorbani, 2014).

### 1.3 Bottom-up method

In the bottom-up technique, nanoparticles are formed by joining smaller atoms and molecules, so this method is also called the building-up method. Laser pyrolysis, spray pyrolysis, and green synthesis are examples of this method. The laser pyrolysis method involves the application of laser energy to form nanoparticles. The precursor absorbs laser energy, such as infrared CO_2_ lasers, to incite homogeneous nucleation reactions (Swihart, 2003). In the spray pyrolysis technique, nanoparticle precursors in a vapor state are directly carried by a nebulizer into the hot reactor. Metals such as nitrate, acetate, and chloride are often used as metal precursors (Okuyama and Lenggoro, 2003).

In general, physical and chemical approaches require high energy consumption, are expensive, and use poisonous and risky chemical agents that are responsible for many hazards to the environment (Nath and Banerjee, 2013). Against those limitations, “green synthesis” techniques are gaining attention in contemporary research into the development of nanoparticles. Green synthesis avoids the production of undesirable or dangerous by-products through the build-up of dependable, eco-friendly, and sustainable synthesis procedures. Additionally, green synthesis is cost-effective and does not involve the use of high energy, temperature, pressure, and toxic chemicals (Singh et al., 2018).

**FIGURE 2:**
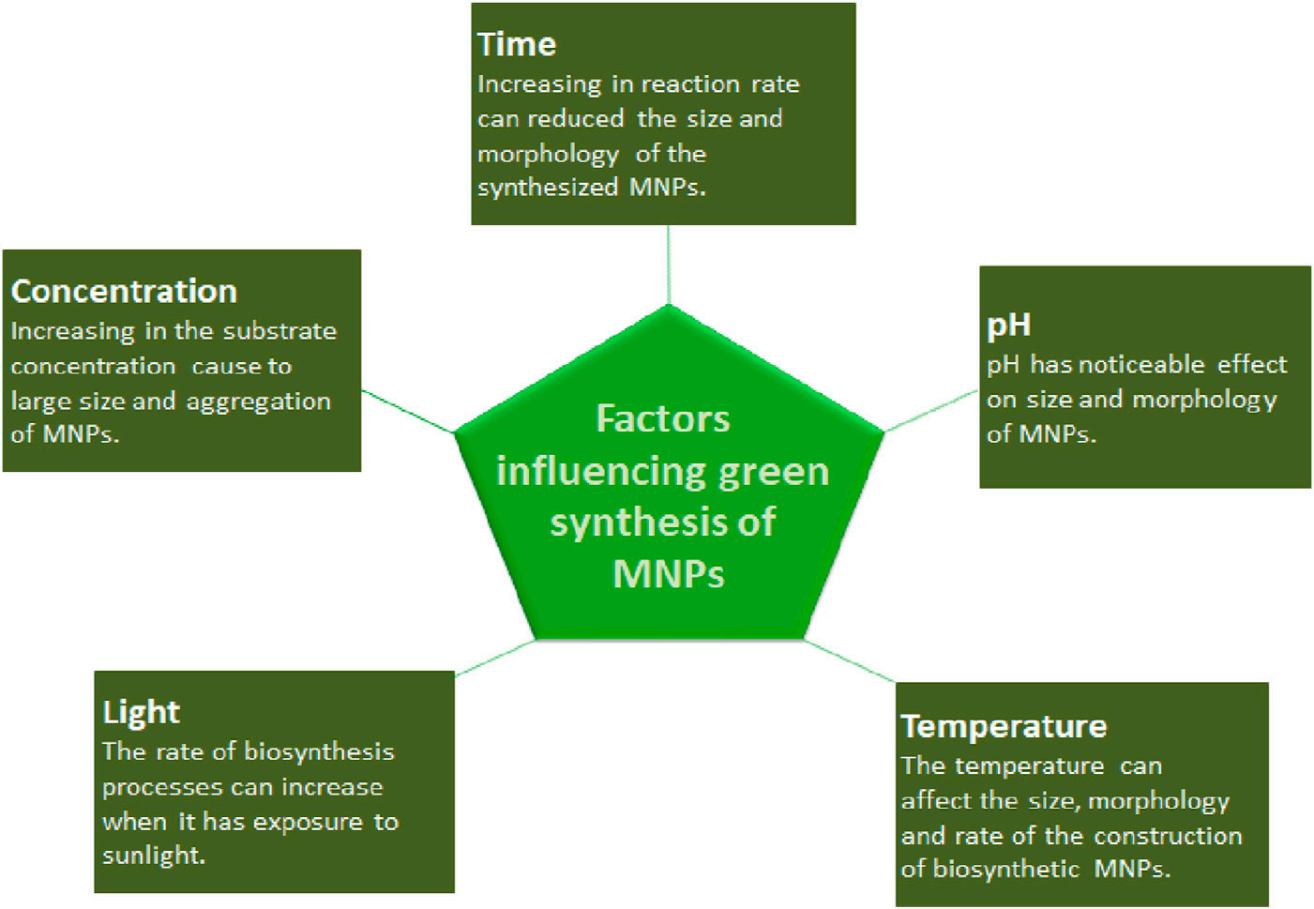
Factors affecting the green synthesis of MNPs.

Microorganisms and various plants can synthesize MNPs by utilizing microbial enzymes, polysaccharides, vitamins, and other biological and biodegradable substrates (Korbekandi et al., 2009). The synthesized nanoparticles from these microorganisms have found applications in different fields and have less toxicity. These properties make MNPs an appropriate option for developing drug delivery systems and as carrier material for sensors in diagnostic devices (Khandel and Shahi, 2016). Biogenic MNPs can be created in two less time-consuming ways: either by combining the metal salt with intra- and extracellular extracts of microorganisms at ambient temperature or by reducing metallic ions to their stable forms where enzymes act as reducing agents (Sadowski et al., 2008; Deplanche et al., 2010). Bacterial species are used extensively in commercial biotechnological applications such as genetic engineering, bioleaching, and bioremediation (Singh et al., 2018). Bacteria can reduce metal ions, and due to their low energy consumption and process controllability, they are a favorable source of synthesizing MNPs (Fang et al., 2019). Among the bacterial species, Actinomycetes and Prokaryotic bacteria have been widely explored for making metal or metal oxide nanoparticles. A single bacteria can alter the toxicity of metal ions into non-toxic, safe NPs. Another important species of microorganisms is fungus. Compared to all other microorganism species, fungus has shown higher productivity and tolerability to metals (Singh et al., 2016). Syntheses that use fungi and yeast can be done via intracellular and extracellular approaches. The expression of mycelia provides a high surface area, which supports the fungus in secreting more proteins than bacteria, which consequently results in a tremendous increase in MNP production. Various types of algae have also been found to be capable of holding heavy metals and, therefore, can also be used to synthesize MNPs of heavy metals such as gold and silver (Shnoudeh et al., 2019). Plants biomolecules, such as proteins, carbohydrates, and coenzymes, may have the potential to reduce metal salt into nanoparticles (Singh et al., 2018). The MNPs from plant extract can be prepared simply by mixing the metal salt solution with the extract of the plant of interest (Malik et al., 2014). The size, surface morphology, and quality aspects of MNPs prepared from plant extract depend on the mixing ratio of plant extract and metal salt and the reaction temperature (Malik et al., 2014). Details of some MNPs synthesized by microorganisms and plants, their characteristics, and indications are summarized in Tables 1, 2, respectively. The rate of green synthesizing of MNPs, their size, and morphology could be managed and modified by manipulating and controlling the parameters including temperature, pH, concentration of substrate, exposure time to substrate (Figure 2) (Punjabi et al., 2015), and light (Raut et al., 2014).

**TABLE 1:** Green syntheses of MNPs from various microorganisms.

Type of microbe	Species	Nanometal	Size (nm)	Morphology	Application	Efficiency of application of NPs	Reference
Bacteria	*Bacillus cereus*	Silver	20–40	Spherical	Antibacterial activity against *Escherichia coli, Pseudomonas aeruginosa,Staphylococcus aureus*, *Salmonella typhi*, and *Klebsiella pneumoniae* bacteria	The synthesized nanoparticles were found to be spherical with a size in the range of 20–40 nm. The endophytic bacteria were able to synthesize silver nanoparticles with potential antibacterial activity	Sunkar and Nachiyar (2012)
Bacteria	*Lactobacillus casei*	Silver	20–50	Spherical	Drug delivery, oncological application, and labeling with biomolecules	This review article shows a successful environmentally friendly nanoparticle synthesis technique	Korbekandi et al. (2012)
Bacteria	*E. coli* DH 5α	Gold	8–25	Spherical	Direct electrochemistry of hemoglobin		Du et al. (2007)
Bacteria	*Micrococcus yunnanensis*	Gold	53		Antibacterial and anticancer	The antibacterial effect of the produced Au NPs and Au^3+^ ions against three Gram-positive and four Gram-negative bacterial strains exhibited lower inhibitory activity of Au NPs than that of Au^3+^ ions	Jafari et al. (2018)
Bacteria	*Shewanella loihica*	Copper	10–16	Spherical	Antibacterial	It is anticipated to provide insights into developing a more potentially integrated platform for precise thrombus therapy.	Lv et al. (2018)
Fungus	*Fusarium semitectum*	Silver	10–60	Crystalline spherical	Biolabeling	Simple biological process for synthesizing silver nanoparticles using the fungus *F. semitectum*, making the downstream processing easier	Basavaraja et al. (2008)
Fungus	*Cariolus versicolor*	Silver	25–75	Spherical	Labels for living cellsand tissues	Controlled and up-scalable route for the biosynthesis of silver nanoparticles (NPs) mediated by the fungal proteins of *Coriolus versicolor*	Sanghi and Verma (2009)
Fungus	*Aspergillus oryzae*	FeCl_3_	10–24.6	Spherical	Agricultural, biomedical, and engineering sectors	Green approach was used to synthesize iron nanoparticles using the fungi *Aspergillus oryzae* TFR9	Tarafdar and Raliya (2013)
Algae	*Sargassum muticum*	Gold Silver	10.4 41	Spherical Spherical	Antibacterial and antioxidant Antibacterial and antioxidant	Gold and silver nanoparticles were synthesized by an extract of the brown macroalga *Sargassum muticum*. Ag@SM showed good inhibitory capacity on Gram+ bacteria, especially on *Staphylococcus aureus,* with an MIC of 3.38 μg/mL	González-Ballesteros et al. (2020)
Yeast	*Candida glabrata*	CdS	20 Å- 29 Å	Hexamer	Physiological	Reported the biosynthesis of quantum crystallites in yeasts *Candida glabrata* and *Schizosaccharomyces pombe*, cultured in the presence of cadmium salts	Dameron et al. (1989)

**TABLE 2:** Green synthesis of MNPs from plant and their extracts.

Plant origin	Nanometal	Size (nm)	Morphology	Applications	Reference
*Aloe barbadensis* Miller	Gold and silver	10–30	Spherical and triangular	Cancer hyperthermia and optical coatings	Chandran et al. (2006)
*Acalypha indica*	Silver	20–30	Spherical	Antibacterial activity against waterborne pathogens	Krishnaraj et al. (2010)
**Apiin extracted from henna leaves**	Silver and gold	39	Spherical, triangular, and quasi-spherical	Hyperthermia of cancer cells and IR-absorbing optical coatings	Kasthuri et al. (2009)
*Coriandrum sativum* **(coriander)**	Gold	6.75–57.91	Spherical, triangular, and truncated triangular decahedral	Drug delivery, tissue/tumor imaging, and photothermal therapy	Narayanan and Sakthivel (2008)
*Medicago sativa* **(alfalfa)**	Gold	2–40	Irregular, tetrahedral, hexagonal platelet	Labeling in structural biology	Gardea-Torresdey et al. (2002), Gardea-Torresdey et al. (2003)
	Iron oxide	2–10	Decahedral and icosahedral Crystalline	Cancer hyperthermia and drug delivery	Herrera-Becerra et al. (2008)
*Sapium sebiferum*	Palladium	2–14	Spherical	Antibacterial activity against *S. aureus*, *Bacillus subtilis*, and *P. aeruginosa*, and photocatalytic activity	Tahir et al. (2016)
*Ocimum tenuiflorum*	Selenium	15–20	Spherical	Medical and pharmaceutical	Liang et al. (2020)

## 2 Characterization techniques of MNPs

**FIGURE 3:**
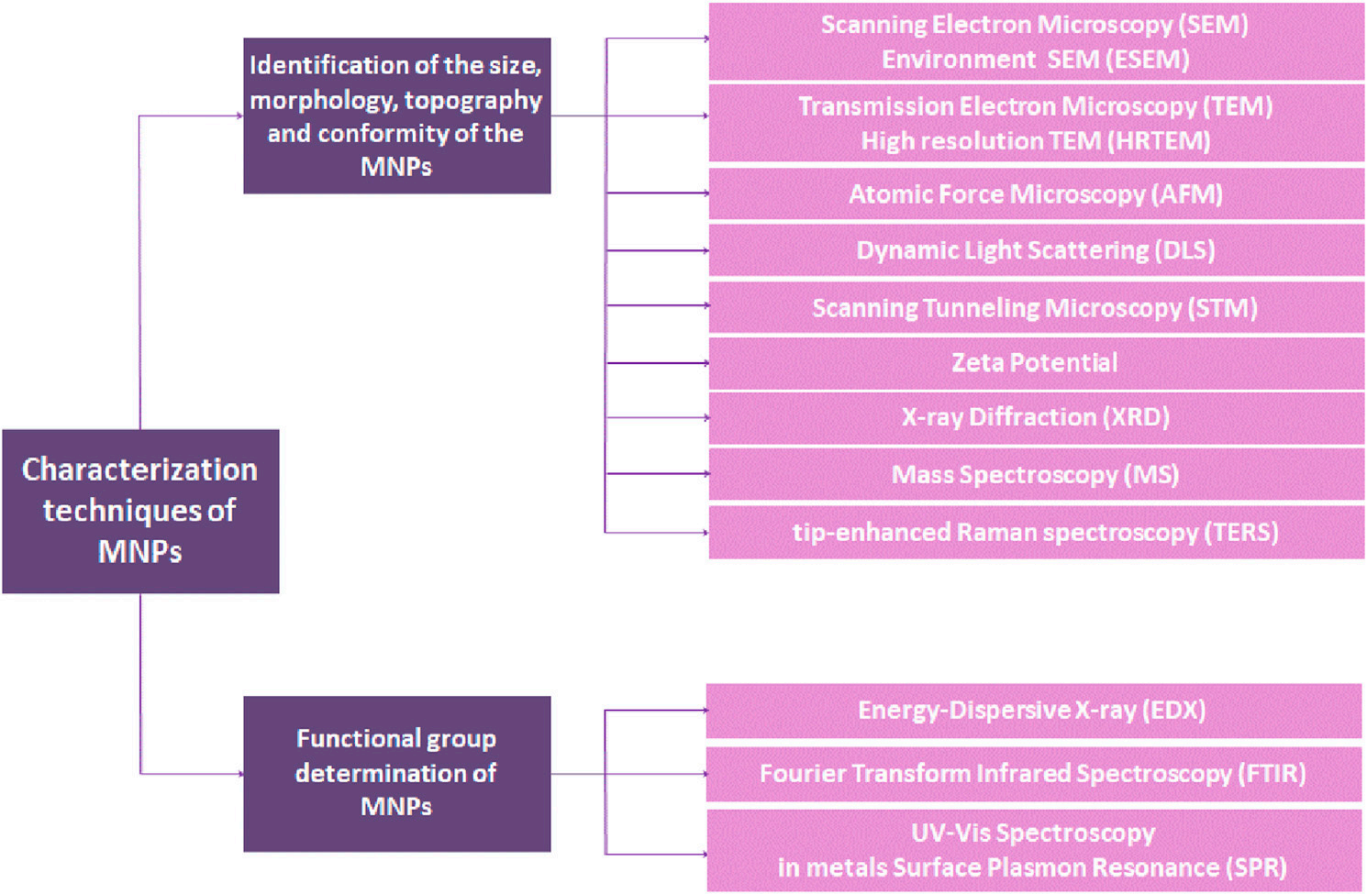
Various methods and the classification for determination of MNPs.

Characterization of MNPs is a crucial step for identifying the nanoparticles according to their size, morphology, dispersity, chemical composition, and surface area (Khandel and Shahi, 2016). Understanding the detailed surface morphological properties of MNPs helps select the most relevant functional groups for surface functionalization in order to find a suitable therapeutic use for MNPs. Two techniques are employed for this purpose: a) identification of the size, morphology, topography, and conformity and b) functional group determination. These categories and their methods are summarized in Figure 3 (Shnoudeh et al., 2019).

### 2.1 Imaging techniques

#### 2.1.1 Optical imaging

Optical imaging is a recent biomedical and clinical imaging strategy for non-invasive monitoring inside the body, and the images obtained offer good cellular resolution. This approach detects photons released by bioluminescent, fluorescent (two-photon *fluorescence* (TPF) and fluorescence lifetime imaging microscopy (FLIM)), or even Raman (RS) probes in the visible and near-infrared (NIR) regions. Unlike other techniques, such as X-rays that use ionizing radiation, optical imaging is regarded as a safer radiation imaging strategy that is perfect for repeating these procedures and is also moderately cost-effective. In addition, optical imaging is significantly amenable to extending over a wide region of wavelength and resolution, and multimodal and optical imaging are generally employed in combination with other imaging methods (Pouratian and Sheth, 2009; Arranz and Ripoll, 2015). Emerging optical imaging strategies, including FLIM, can be employed to monitor the cellular uptake of theranostic (therapeutic and diagnostic) nanoparticles and drug release. Optical imaging has some limitations, including superficial tissue penetration, background noise because of the auto-fluorescence of tissue and protein, and other component absorption at different wavelengths.

Huang et al. (2013) developed a versatile theranostic platform for early-stage tumor detection and targeted orthotopic glioblastoma based on multifunctional mesoporous silica nanoparticles for delivering mesenchymal stem cells (Huang et al., 2013). Song et al. (2017) enhanced the physico-optical properties of branched nanoporous gold nanoshell (BAuNSP) for optical imaging and cancer therapy by controlling the porosity and roughness. A rather novel optical imaging approach operates on light-scattering principles like Raman spectroscopy. The Raman effect is relatively weak and requires generating a plasmon resonance by a surface of metallic nanoparticles (MNPs) that expands the electromagnetic field and improves the Raman signal by multiple orders of magnitude. Similar optical approaches have been reported for photothermal therapy and surface-enhanced Raman scattering (SERS) imaging (Wen et al., 2019; Tabish et al., 2020; Sarbadhikary et al., 2021; Sun et al., 2017) including real-time monitoring and releasing the polydopamine from gold nanorods (AuNRs) under near-infrared (NIR) laser irradiation (Sun et al., 2017). In the new study, researchers developed a novel *in-vivo* X-ray excited optical luminescence (XEOL) imaging agent for deep tissue visualization based on europium-doped TaOx nanoparticles (Narasimhan et al., 2021).

#### 2.1.2 Magnetic imaging

##### 2.1.2.1 Magnetic resonance imaging (MRI)

MRI is a safe and non-invasive imaging method that produces images based on nuclear magnetic resonance (NMR) of the relaxation properties of hydrogen nuclei (in water and fat) in a strong applied magnetic field. MRI has a suitable spatial resolution but limited sensitivity. However, this imaging method has been used in theranostic applications such as tracking cellular uptake and cell therapy to monitor drug release and its effects on the environment. The application of MRI using nanoparticles is a promising tool for researchers and clinicians, allowing them to monitor the progress of peripheral arterial disease (PAD) effectively via the inclusion of imaging agents, which are classified as T1 and T2 shortening agents. The most commonly used nanoparticle in this method in the former stage is gadolinium (Gd), a contrast agent that can brighten MRI images, while the latter revolves around iron oxide nanoparticles. T2 shortening agents such as magnetic iron oxide (Fe_2_O_3_ and Fe_3_O_4_) participate in this imaging tool by facilitating extravasation within the vascular tissues, where they are taken by macrophatic cells (Ahrens and Bulte, 2013; Serkova, 2017). Guo et al. (2012) described a T-cell–targeted theranostic platform based on SPION (superparamagnetic iron oxide nanoparticles) for detecting acute allograft rejection after an allogeneic heart transplant.

PEGylated-DOX-gadolinium-gold nanorods were developed as a new theranostic complex by Khan et al. (2019) that can provide multifunctional features. This nanoplatform improved the tumoral toxicity in human pancreatic cancer cell lines (MIAPaCa-2) compared to doxorubicin. Furthermore, an enhanced permeability retention (EPR) effect in MRI enables positive contrast for imaging and an adjusted size for passive targeting of tumor sites (Khan et al., 2021). Similarly, a multifunctional theranostic system based on doxorubicin-loaded onto PEGylated gadolinium nanoparticles (Gd-NP) and graphene oxide (GO) functionalized by folic acid (FA) exhibited very good photothermal-chemotherapeutic efficacy targeting effects and diagnostic ability of the tumor site (Shi et al., 2016).

Metallic theranostic nanoplatforms promising therapeutic and bioimaging properties provide a real practical therapeutic entity in the biomedical field (Korolev et al., 2021). Janus metallic nanoplatforms, with their unique optical, thermal, magnetic, and electric structures and properties, are also considered for theranostic nanoplatforms based on a stimuli-responsiveness manner (Shao et al., 2021).

##### 2.1.2.2 Magnetic particle imaging (MPI)

Magnetic particle imaging (MPI) is a tomographic technique that can provide the 3D distribution of superparamagnetic iron oxide nanoparticles (SPIONs). Unlike traditional bioimaging methods, MPI overcomes the limitations of tracking contrast elements or *in vivo* tracers. The signal produced by MPI is derived primarily from nanoparticles rather than surrounding tissue (Borgert et al., 2012; Panagiotopoulos et al., 2015). Therefore, mixing MPI with an additional tomographic method permits the registration of nanoparticle signals and other anatomical data, which improves the diagnostic potential. In the external magnetic field, MNPs are extremely saturated and magnetized. In the same way, when SPIONs placed in the field-free region (FFR) are exposed to magnetic fields, the particles continuously oscillate (Talebloo et al., 2020). To generate a signal, the FFR crosses a place containing the particles, and the magnetization changes in response. MPI presents perfect image contrast because that does not indicate noise from the background tissues. In addition, there is no signal debilitation of depth in tissue, permitting bioimaging of depth inside the organs quantitatively.

Recent investigations have indicated the ideal possibility of MPI for radiation-free, very sensitive vascular bioimaging and cellular tracking (Zhou et al., 2018). An *in vivo* study by Mohtashamdolatshahi et al. presented a new MPI agent focusing on multicore nanoparticles (MCP 3). Their results demonstrate that the nanoplatform provides suitable quality images even at a lower dosage (Mohtashamdolatshahi et al., 2020). In a novel study by Irfan et al., NiFe_2_O_4_ coated and functionalized with citric acid and PAA (polyacrylic acid) were introduced as bioimaging MPI platforms with high contrast and low relaxation time (Irfan, Dogan, Bingolbali, and Aliew, 2021). Jiang et al. developed a mixed metal metal-organic platform based on ZnFe_2_O_4_/C@PDA (SPIONs supported by carbon) with high biocompatibility that can act as high-performance bioimaging agents (Jiang et al., 2021). Chen et al. (2014) reported a simulation of the reconstruction of MPI agents with high accuracy (Chen et al., 2022).

### 2.2 Ultrasound imaging

Ultrasound is a safe, cost-effective, and fairly fast imaging method. A transducer held against the skin emits sound with high-frequency waves, and the reflected echoes record and make images of internal organs and tissues. To acquire good-resolution images of tissues that are placed in the depths of the body, the contrast agents that produce gas can be employed to enhance imaging as the gases contain dissimilar acoustic properties from tissues or can motivate the environment with various acoustic properties (Moran and Thomson, 2020; Jani et al., 2021). This method is rather invasive. 

In the field of photodynamic therapy (PDT), Gao et al. published a research article on the use of ultrasound imaging to monitor oxygen-generating MnO_2_ nanoparticles (MnO_2_ NP). They used the reactivity of MnO_2_ NP to H_2_O_2_ and extended oxygen-generating, targeting to take account of the sufficient generated oxygen in the tumor site before starting photodynamic therapy. To improve ultrasound imaging, they created an ICG-HANP/MnO2 (IHM) nanocomplex by encapsulating a MnO2 NP in hyaluronic acid (HA) functionalized with indocyanine green (ICG). Moreover, the production of oxygen was seen with constant NIR laser irradiation for 10 h and increased cellular toxicity in SCC7 tumor-bearing rodents (Gao et al., 2017).

Another new theranostic strategy developed by Zhang et al. is based on a metal-organic framework as a dual-sonosensitizer nanoplatform in sonodynamic therapy (SDT). SDT exhibits excellent cell and tissue penetration with minor radiation harm to normal cells and tissues, making it a potential cancer therapeutic method. The Zr-MOF@AIPH nanoplatform is designed by loading an alkyl radical generator (AIPH) onto a zirconium metal-organic framework and can decrease the cavitation threshold, improving the acoustic cavitation effect, thereby facilitating penetration of that at the tumor site (Zhang et al., 2022). A novel class of sonosensitizer designed by a plain method, MnCO_3_ NPs, was employed to enhance SDT by Zhang et al. Their results showed that the CO_2_ bubbles induced necrosis in cells by ultrasonic cavitation and were employed for imaging. In addition, the *in vitro* experiments demonstrated increased rates of tumor inhibition due to triggering the mitochondrial pathway of apoptosis (Zhang et al., 2021). A novel biodegradable nanocomplex (CSI) was developed by catalase (CAT) encapsulation into SiO_2_ nanoparticles (CAT@SiO_2_) and ICG as a sonosensitizer for glioblastoma treatment. Moreover, to cross the blood–brain barrier, the CSI was coated with macrophage-derived exosomes (CSI@Ex) that were functionalized and modified with AS1411 aptamers (CSI@Ex-A) (T. Wu, Liu, Cao, and Liu, 2022).

### 2.3 Nuclear imaging

Nuclear bioimaging techniques such as single-photon emission computed tomography (SPECT) and positron emission tomography (PET) involve injecting radiolabeled tracers, radionuclides, into the body to investigate physiological functions. To acquire 3D images, gamma rays generated by the injected radionuclide agents are collected by a gamma camera (Brant and Helms, 2012). For decades, SPECT imaging has been a promising bioimaging method in nuclear medicine clinics. It uses low-cost radionuclides like technetium-99m (99 mTc), which can be extracted fresh from generators of molybdenum-based technetium and given to clinics on a regular schedule. 99 mTc offers various advantages for nuclear imaging, such as a half-life (approximately 6 h) and a 140 keV emission of gamma-ray, which is excellent for the scintillation crystals used in most clinic gamma cameras. PET is a method of nuclear imaging that employs positron emission to produce two 511 keV gamma-ray (high energy) photons that provide quantitative data (Shooli et al., 2019; L; Zhao et al., 2018).

Tsoukalas et al. developed a dual-manner nanoplatform (Fe_3_O_4_-DMSA-SMCC-BCZM-^99m^Tc) based on modified and coated iron oxide nanoparticles for *in vitro* targeted SPECT and MRI of vascularization at the tumor site. Bevacizumab (BCZM) was used as a tumor angiogenesis inhibitor, and dimercaptosuccinic acid (DMSA) was offered as a functionalized group for improved biocompatibility. Initial imaging examinations proved the potential of their nanoplatform for targeted dual-manner imaging. Moreover, the results indicate that the nanoplatform could be a significant diagnostic tool for bioimaging and a good candidate for cancer theranostic applications (Tsoukalas et al., 2018).

Metal–phenolic nanoparticles (MPNs) have attracted attention in bioimaging owing to their versatility in constructing predesigned forms with special properties. Before MPNs can be used in biomedical applications, they must be optimized in selective applications to eliminate their drawbacks, such as accumulation in non-selective tissues and toxicity. Suárez-García et al. fabricated a metal–phenolic nanoplatform for *in vivo* SPECT/PET/CT imaging in tumor-bearing rodents. Their results demonstrated that polyphenols for the fabrication of hybrid nanoplatforms can be developed flexibly for effective use in bioimaging (Suárez-García et al., 2021).

### 2.4 Computed tomography

Computed tomography (CT) is an accepted and widely used technique that permits tissue imaging and provides complementary anatomical visualization alongside other imaging techniques, including SPECT and PET. CT can provide non-invasive, three-dimensional anatomical data on particular tissues, including cardiovascular, liver, gastrointestinal, and lung. One weakness of this method is its lack of sensitivity to contrast agents compared to other methods like MRI (Y. C. Dong et al., 2019). Yang et al. (2018) introduced a functionalized nanoplatform based on bismuth nanoparticles (BiNPs) as a feasible photothermal therapy (PTT) agent to be monitored by photoacoustic (PA) and CT imaging. Gao et al. presented a novel type of nanocage based on Au@PEG (AuNC@PEGs) with a great absorption coefficient for X-rays. The gold nanocages act as a contrast agent for CT scan imaging, and their results indicated that AuNC@PEGs maintained suitable dispensation in the aqueous phase, good biocompatibility, and great ability for X-ray absorption. Moreover, *in vivo* investigations have demonstrated that the AuNC@PEGs have an obvious contrast gain, prolonged circulation time, and poor toxicity. Thus, the functionalized AuNC@PEGs have excellent potential for biomedical application and are promising contrast agents in CT scan imaging (Y. Gao et al., 2020).

### 2.5 Hybrid bioimaging technique

Among the currently available imaging techniques, nuclear imaging, including SPECT and PET, presents an extremely sensitive and nano-invasive approach for quantitative analysis at the molecular level of physiological functions. However, these methods provide mainly practical data that may not directly relate to anatomical structures. The lack of high-contrast information in nuclear image information is a significant limitation of imaging techniques such as SPECT and PET (Cal-Gonzalez et al., 2018; Luengo et al., 2021). Better and more accurate images can be obtained of both soft and hard tissues using a combination of bioimaging methods (Shan et al., 2022).

Evertsson et al. developed the MMUS imaging technique based on a novel ultrasound approach that employs SPIONs as a contrast agent for hybrid imaging. The sentinel lymph node (SLN) rodent model was selected to evaluate the integrated magnetomotive ultrasound PET/CT and MRI 1 hour after an injection of ^68^Ga-labeled superparamagnetic iron oxide nanoparticles, and their results indicated that the nanoplatform provided feasible contrast enhancement (Evertsson et al., 2017). Moreover, SPIONs and Zr were employed as a T1/T2 dual-mode platform for hybrid PET-MRI imaging. The combination of PET and MRI indicates the remarkable sensitivity of spatial resolution and provides excellent contrast in soft tissues (Gholami et al., 2020).

Tran-Gia et al. examined the accuracy of quantitative hybrid Lu-177 SPECT and CT bioimaging in the MRT Dosimetry project (Tran-Gia et al., 2021). The combination of SPECT and CT can enhance the accuracy of sentinel node biopsy (SNB) for melanoma diagnostics. The benefit of hybrid imaging has implications for surgical procedures and pathology services with improved diagnostic and treatment (Moncrieff et al., 2022).

## 3 Application of theranostic MNPs

Recently, MNPs have been used in several biomedical applications owing to their various physicochemical properties, suitable preparation methods, stability, and acceptable biocompatibility. MNPs can interact with the magnetic fields and even alter those in their vicinity, therefore elevating MRI. The external magnetic fields can generate various forces that result in the rotation, dissipation, and translation of energy. These phenomena indicate many applications in the cellular study of separation and biomarkers, delivery of protein cargo and targeted drugs using magnetism, stimulation of receptors on the cell surface through magneto-mechanical and biomedical theranostic applications, drug release triggers, and hyperthermia. Their usage in biomedical investigation depends on their properties, and the most important aspect is their biocompatibility (Wabler et al., 2014; Farinha et al., 2021).

### 3.1 Cancer

Because of their unique physicochemical properties, metallic nanoparticles (Au-based, Ag-based, and oxide-based), such as zinc oxide and iron oxide, have been widely used in biomedicine for a variety of applications, including bioimaging, drug and gene delivery, and theranostic. Lately, the biosynthesis of MNPs using green synthesis techniques has earned enormous attention owing to its considerable advantages over conventional techniques, which are discussed in many articles. Biosynthesized MNPs such as AuNPs and AgNPs have indicated potential practical application in anticancer drug delivery *in vitro* and *in vivo*. Several published reports of biosynthesis AuNPs conjugated with doxorubicin (Dox) and 5-fluorouracil have demonstrated a significant decrease in melanoma and breast tumor growth in rodent models (Ganeshkumar et al., 2013; Patra et al., 2015; Mukherjee and Patra, 2016). Moreover, the *in vitro* and *in vivo* anticancer activities of Dox-loaded DNA/AuNPs have shown superior results by inhibiting tumor cell growth in different human ovarian cancer cell lines, including SK-OV-3, HEY A8, and A2780 (Lee et al., 2020). In another article by Arkaban et al., a carrier for targeted imaging and therapeutics integrated with folic acid was introduced to deliver Dox conjugated AuNPs-based nanocomposite AuNPs@MnCO_3_/Mn_3_O_4_ coated with polyacrylic acid for capturing tumors in a targeted manner. The system performance is introduced as a therapeutic targeting and MRI contrast agent with a high capacity for doxorubicin delivery and high interaction with the folate receptor in breast cancer cells (Arkaban et al., 2021).

### 3.2 Bacterial

Antimicrobial resistance has increased the need to investigate novel and innovative approaches to antimicrobial therapy. Various innovative strategies under research include antimicrobial peptides (AMPs), antibodies, the antimicrobial activity of NPs, and phage therapy. Lately, MNP-based approaches have been extended to infections induced by drug-resistant bacteria and biofilms. The MNPs can destroy microbes through three essential mechanisms, including disrupting the cellular plasma membrane, releasing toxic ions, and generating reactive oxygen species (ROS) that interfere with bacteria components (Annunziato, 2019; León-Buitimea et al., 2020). Iron oxide nanoparticles, the leading class of MNPs, have some key significance owing to their magnetic and catalytic properties.

Biofilms are communities of bacteria that stick to different surfaces and are entrenched in a matrix of extracellular polymeric substances (EPS). Because of the broad antimicrobial blockage caused by EPS, biofilm treatment with conventional antibiotics is difficult (Xu, Akakuru et al., 2019). In other words, EPS is known as a local barrier. The EPS is like a bacterial shield and prohibits the diffusion of drugs, thereby resulting in bacterial resistance and making biofilm removal a difficult task. IONPs, owing to their paramagnetism properties, are widely used for developing magnetic microrobots. Moreover, they show unique properties such as peroxidase-like, which implies that the Fe in the structure can go forward to the Fenton reaction, inducing OH that destroys the matrix of biofilms (Cheng et al., 2021).

Nowadays, photothermal therapy (PTT) is regarded as a favorable and efficacious antibacterial method owing to its near non-invasiveness, in-depth penetration of tissue, easy operation, and absence of resistance (Behzadpour et al., 2019). Multimodal PTT is recognized as a beneficial strategy owing to short-time irradiation, decreased dose, and enhanced performance. Liu et al. reported a multifunctional magnetic nanoplatform based on copper ferrite nanoparticles that were functionalized with hemoglobin (Hb-CFNPs) (Liu, Luehmann et al., 2019). Due to the effective capability of light absorption, photoresponsive nanoplatforms can create heat that can destroy bacteria by interrupting bacterial cell membranes and inducing the denaturation of proteins, which causes cell death (Ren et al., 2020).

### 3.3 Vascular and arterial diseases

Cardiovascular disease (CVD) is the main cause of death worldwide. CVD is related to the heart and blood vessels, including myocardial infarction (MI), ischemic injuries, atherosclerosis, and thrombosis. Among CVD disorders, atherosclerosis is the most prevalent cause associated with inflammation of the blood vessels (Gaziano et al., 2006). The leading challenges in the diagnosis and treatment of these kinds of disorders are related to pathophysiological complexity. Theranostic nanoplatforms have been developed to eliminate the limitations of recent diagnostic and treatment strategies. The molecular mechanisms of CVD disorders have been extensively studied, and methods for developing specific nanoplatforms targeted at damaged cells have been developed (Brewer et al., 2015; Wu et al., 2021).

Cerebral venous thrombosis (CVT) was considered a rare disease; however, new research suggests that it is more common than previously thought. A recent retrospective population-based study from Adelaide, Australia, found an incidence of 15.7 cases per million per year, which is higher than previous estimations of 2.5 per million per year. It has been found by the increased application of more widely available imaging techniques for these diseases. It involves both pediatric and adult populations, with a higher risk among women of childbearing age (Devasagayam et al., 2016; Kim et al., 2017; Luo et al., 2018). Clinical diagnosis is often difficult, as it can present with a plethora of signs and symptoms, mimicking numerous neurological pathologies. The radiologist plays a key role and may be the first to suggest the diagnosis. The usage of MNPs can be reduced remarkably as the researchers designed EGFP-EGF1-NP-Fe_3_O_4_ for an earlier diagnosis of cerebral thrombosis by taking advantage of the EGFP–EGF1 fusion protein. In this method, EGF1 can bind with tissue factor and enhance green fluorescent protein, which has previously been widely used as a fluorescent protein marker. In 1 h, the concentration of EGFP-EGF1-NP-Fe_3_O_4_ or NP-Fe_3_O_4_ reaches its maximum in the infarction areas (Hu, Fan et al., 2019). Based on another study, TAP-SiO_2_@AuNPs were successfully accumulated in the thrombus by their particle size-dependent capturing property, and they presented a potential X-ray absorption property in a dose-dependent manner. Finally, the thrombotic lesion was clearly distinguished from peripheral tissues by dual NIRF/micro-CT imaging after intravenous injection of TAP-SiO2@AuNPs in the *in situ* thrombotic mouse model simultaneously. This study showed that thrombin-activatable fluorescent peptides incorporating silica-coated gold nanoparticles could potentially be used as a dual imaging probe for direct thrombus imaging and therapy in clinical applications (Kwon et al., 2018; Lv et al., 2022).

Quantum dots are fluorescent semiconductor NPs that contain group II-VI (e.g., CdSe and CdTe), III-V (e.g., InP and InAs), IV-VI (e.g., PbTe and PbSe), or I-III-VI (e.g., CuInS_2_) elements. They are characterized by a narrow and symmetric emission band (∼30 nm) that can be tuned precisely by changing the NP sizes and compositions. The broad absorption spectra and large Stokes shifts of quantum dots allow simultaneous imaging of multiple types of quantum dots with single wavelength excitation. Quantum dots also have the capability of coupling to a biomolecule for targeted imaging. For cerebrovascular thrombosis detection, one study used lead sulfide quantum dots with 1100 nm emission peaks, which can be used in NIR fluorescence imaging. This was tested in septic mice, and the results showed it to be a useful tool for evaluating the pathological state of cerebral blood vessels in septic mice. The results introduced short-wavelength infrared region-emissive indium arsenide quantum dots with high-resolution multicolor imaging that are readily modifiable, provide deep penetration, and have fast acquisition speed in small-animal models. These dots could simultaneously quantify the metabolic turnover rates of lipoproteins in several organs.

Peripheral arterial disease (PAD) mainly occurs in the lower extremities and is characterized by chronic narrowing of the arteries with occlusion and/or loss of functionality. The endothelial wall lining and the native extracellular matrix (ECM) architecture are often damaged, leading to a narrowed vessel and insufficient delivery of oxygen-rich blood to organs. With its crucial and emerging uses in the prevention, diagnosis, and treatment of several diseases, nanotechnology has shed light on improving health globally for PAD patients, including morbidity and mortality rates (Serkova, 2017; Zemaitis et al., 2017). Commonly, PAD detection techniques produce images of changes in tissue appearance but cannot determine the state of disease. We need brand new, improved, and more accurate diagnostic strategies for PAD, especially for identifying the various stages of this disease (Roy et al., 2018).

In this context, nanotechnology plays a crucial role through the use of very small structures, often less than 100 nm, to effectively localize and confirm events occurring in ailing vessels. Diseased vessels are usually characterized by lipid retention, the expression of cellular adhesion molecules, the destruction of endothelial cells, the production of macrophages, and the formation of plaque. Thus, during the different stages of inflammation, these characteristics create potential molecular imaging probes for detecting PAD.

Molecular imaging is a subset of imaging that involves the depiction and evaluation of any biological process at the cellular and subcellular levels via the application of imaging modalities, including cardiac magnetic resonance molecular imaging (CMRI), CT, optical coherence tomography (OCT), nuclear techniques, MRI, and fluorescence (Talebloo et al., 2020; Teuho et al., 2020). Consequently, these noninvasive imaging strategies provide the visualization of cells and subcellular components ranging from angstroms to centimeters in any living organism. Unlike the limitations of the traditional approach to diagnostics using bare contrast agents, such as poor half-lives and high toxicity levels, researchers incorporate these imaging probes into nanoparticles to overcome these problems (Teuho et al., 2020; Y; Zhang et al., 2014).

Most MNPs used for PET are produced from chelate materials. This chelation can help improve the tracking of NPs. To provide higher sensitivity and better resolution of atherosclerosis progression, a copper comb-like NP labeled with the peptide DAPTA (64CU-DAPTA-comb) was explored, and the results confirmed that it was an effective imaging probe for atherosclerosis (Liu, Guo et al., 2019). In a recent study, the multimodal 64CU-RGO-IONP-PEG nanoparticles (68 ± 7 nm) were assessed for better quantitative analysis of PAD detection. Administration of 64CU-RGO-IONP-PEG nanoparticles in hindlimb mouse models resulted in increased nanoparticle accumulation over time in the ischemic hindlimb compared to the minimal signal observed in control limbs (nonischemic), as confirmed by PET data. Photoacoustic signals also revealed an increase in the ischemic hindlimb 3D post-NP administration compared to the minimal signal from the control limb (nonischemic). Therefore, photoacoustic images provided anatomical characteristics with the highest photoacoustic signaling in the ischemic limb (England et al., 2016; Agrawal et al., 2021).

### 3.4 MNPs engineered for theranostic applications across the blood–brain barrier (BBB)

MNPs have various biomedical applications that are helpful in diagnosis and therapeutic processes, such as neurodegenerative diseases detected and targeted by MNPs, as shown in Table 3. MNPs such as quantum dots (QDs), AuNPs, and magnetic NPs have many advantages in treatment as drug vehicles and imaging agents (Zhu et al., 2017; Khizar et al., 2021). Their physicochemical properties, including good stability, reactivity, and the photothermal and plasmonic characteristics of MNPs, make them suitable agents for theranostic applications. Therefore, AuNPs have been employed in photothermal therapy (PTT) to eliminate the growth of brain tumor cells and cause apoptosis. QDs and MNPs have been employed for bioimaging aims (Carvalho et al., 2019; Khan et al., 2021). Some MNPs have been employed in theranostics owing to their diagnostic and therapeutic applications. Engineered MNPs can be produced by modifying the surface, modulating the size distribution and morphology, and interacting with different ligands; these modifications allow them to be utilized in different brain disease treatments. These modified MNPs with extremely small sizes can pass through the BBB. MNPs are enabled to target tumor cells in particular. Many investigations show that MNPs conjugated with transferrin can cross the BBB, and they are practical in the treatment of glioblastoma. Moreover, IONPs and ferritin conjugation (magnetoferritin) have been identified as crossing the BBB and have also been employed against brain cancers (Fan et al., 2018; Hadadian et al., 2021).

**TABLE 3:** Some neurodegenerative diseases detected and targeted by MNPs.

Brain disease	Nanoplatform	Application	Therapeutic agent	Functionalize group	References
Alzheimer	AuNPs	Imaging therapy	L- and D-glutathione	Peptide chirality	(Zhao et al., 2022; Hou et al., 2020)
	AgNPs	Therapy	*Lampranthus coccineus* and *Malephora lutea F.* Aizoaceae		Youssif et al. (2019)
	IONPs	Imaging and Alzheimer’s disease treatment		Hyaluronic acid (HA)	Chen et al. (2022)
	SPIONs		Nerve growth factor (NGF)	Nerve growth factor (NGF)	Katebi et al. (2019)
Parkinson	Au-TiO2	Detection	Primary antibodies	Primary antibodies	
	AuNPs	Therapy	pDNA incorporated exogenous interfering RNA (RNAi) and nerve growth factor (NGF)	Nerve growth factor (NGF)	Hu et al. (2018)
	AgNPs	Citrate cap			Gonzalez-Carter et al. (2017)
	IONPs	Therapy		Dextran	Chung et al. (2018)
	SPIONs		Nerve growth factor (NGF)	Nerve growth factor (NGF)	Katebi et al. (2019)
Neurotoxicity	Magnetic NPs (Fe^2+^ and Fe^3+^ ions)	Imaging Neuro-protection	Brain-derived neurotropic factor (BDNF)		Pilakka-Kanthikeel et al. (2013)
Multiple sclerosis	Au nanocrystal	Therapy			Clene-Nanomedicine (2021)
Cerebral ischemia	AuNPs	Imaging		Glycol-chitosan/fibrin-targeting peptide	Kim et al. (2015)
	IONPs	Imaging/targeting		RGD peptide targeting αvβ3 integrin Pan-caspase inhibitor	Wang et al. (2018);(Saito et al., 2016)
	SPIONs	Imaging		Rhodamine-B and platelet membrane	Meng et al. (2017); He et al. (2021)
Epilepsy	SPIONs-SiO_2_	Therapy-imaging	Ethosuximide (ESM)		Huang et al. (2009)
	SPIONs	Therapy-imaging	Anti-IL-1β monoclonal antibody (mAb)	Anti-IL-1β monoclonal antibody (mAb)	Fu et al. (2016)

One of the major drawbacks of MNPs is their nanotoxicity in the brain, leading to exacerbated BBB leakage. BBB permeability (BBBP) is one of the main processes in stroke development and, as such, is one of the main targets to account for in NP development. In this context, it is essential that NPs do not make blood vessels leakier than they already are; this might happen in the initial or final phases of stroke. Furthermore, this phenomenon causes the NPs to later return to circulation, leading to hemolysis and the already mentioned platelet aggregation, among other adverse events (Kong et al., 2012; Israel et al., 2020).

MNP nanotoxicity may affect neuron function and impair neurorepair and neurogenesis during the subacute and chronic phases. Oxidative stress due to free radical production could not only lead to neuroinflammation but also to subsequent apoptotic mechanisms, mitochondrial damage, and, eventually, neuronal death. These harmful effects have been noted in different types of NPs (Picca et al., 2020). The metal-based NPs are prone to being the most cytotoxic. Constraints with metal-based NPs are particularly important in the context of stroke because of their ability to cross the BBB and reach the brain tissue. Their applications in clinical imaging have generated interest in their use as drug delivery systems and diagnostic tools in this pathology. Due to their small size and specific physicochemical features, metal NPs show a greater accumulation in the brain and induce higher toxicity than larger NPs (Chenthamara et al., 2019; Dong et al., 2020). Moreover, metal-based NPs can release metal ions due to their dissolution, further exacerbating their toxicity. For example, AuNPs can accumulate in the brain, inducing neurotoxic effects, increased seizure activity, cognition defects, and astrogliosis (Johnsen et al., 2018). Novel AuNP surface modifications for theranostic applications have been based on targeted exosomes (Khongkow et al., 2019). IONPs, which are very important in stroke diagnosis through imaging, can interact with the brain's cellular components. Depending on the presence, chemical composition, and charge of the surface coating, they have the potential to cause changes in synaptic activity, leading to neuroinflammation, apoptosis, and immune cell infiltration. Consequently, a lack of information on the potential neurotoxicity effects of MNPs makes their clinical translation more difficult (Akbarzadeh et al., 2021).

### 3.5 Gene drug delivery and therapy

Gene delivery is an approach to delivering genetic materials, including exogenous DNA or RNA, for therapeutic purposes. The viral vectors chiefly activate the immune system, decreasing the efficiency of gene therapy (Cevher et al., 2012; Shahlaei et al., 2020; Sung and Kim, 2019). These problems can be limited to using non-viral vectors, such as metallic nanoparticles. Several researchers have investigated the possibility of metallic nanoparticle platforms to enhance the effectiveness of immunotherapies. Recent investigations indicate that various shapes of gold nanoparticles (AuNPs) can prevent DNA or RNA from nuclease degradation (Ding et al., 2014; Franco et al., 2015). For example, gold nanoparticles (AuNPs) conjugated to oligonucleotides display special properties that can make them promising gene regulation agents (Tian et al., 2018; Rosi et al., 2020). The AuNPs can be divided into non-covalent and covalent (thiol-functionalization) (Mahato et al., 2019). The covalent AuNPs are capable of activating the genes that are related to immune systems (immune-related genes, or IRGs) in peripheral blood mononuclear cells (PBMC) (Kim et al., 2012). Photothermal therapy (PTT) with AuNPs and gene therapy was found to regulate the nuclear factor kappa (NF-ҡβ) signaling pathway at the tumor site (Ahn et al., 2017; Al-ofi & Al-Ghamdi, 2019). Tumor cells and tumor-associated macrophages treated with siRNA inhibited VEGF expression, resulting in tumor regression (Xin et al., 2017; Yang et al., 2021). In addition, the quantum dots (QD) can be conjugated with genetic materials such as oligonucleotides (carboxylic acids as functional groups) and targeted toward DNA or mRNA. QDs can be loaded with genes associated with the drugs (encapsulation or surface interaction) so that QDs can protect the gene against nuclease degradation (Banerjee et al., 2016; Shahmuradyan and Krull, 2016).

Several *in vitro* investigations have been conducted in conditions that do not reflect the physiological conditions to evaluate MNP toxicity. The size of MNPs is important in determining their cytotoxicity, which depends on the surface of MNPs, including the generation of ROS. The toxicity of metallic nanoparticles is generated by ROS on cells, the generation of which can be motivated by different MNP action mechanisms (Yao et al., 2019; Mehta et al., 2021). Functional groups such as prooxidative on the MNP surface, the redox cycle, and the interaction of MNPs and cells are the major aspects of generating ROS. In addition to their surface characteristics, MNPs can operate as ROS generation catalyzers through a variety of methods. Immunocompetent cells see MNPs as a hazard and respond by generating ROS as a defense mechanism. As signal transducers, MNPs can stimulate cellular pathways involved in ROS generation, just as transition metals can trigger ROS generation by participating in Fenton and Haber–Weiss processes (Chawla et al., 2019; Canaparo et al., 2021). Electron leakage occurs when MNPs interrupt the mitochondrial electron transport chain, which allows free electrons to create ROS from oxygen particles (Gallud et al., 2019).

The release of components of MNPs as free ions, which might have toxic effects, is another critical activity of MNPs on cells. For example, AgNPs disrupt the growth of medaka (*Oryzias latipes*) and Danio (*Brachydanio rerio*) larvae (Kwok et al., 2016; Qiang et al., 2020). Various genes were up- and downregulated in zebrafish embryos treated with silver nanoparticles (AgNPs) and silver nanotubes (AgNTs). From a total of 264 genes, AgNPs induced the downregulation of 166 genes and the upregulation of 98 genes. AgNTs caused 139 downregulated genes and 36 upregulated genes out of 175 genes. The most upregulated genes caused by the AgNPs are cellular component (zgc: 175127), metal ion binding gene (zgc: 114104), lipid and carbohydrate metabolisms (sort1b and idh3g, respectively), proteolysis (caspb), developmental processes (thrab), lectin (lgals3l), and pvalb5 (calcium/calmodulin-binding and notch pathway gene) (Kohan-Baghkheirati and Geisler-Lee, 2015; Lee, Horng et al., 2019). The cytotoxicity and significant effect on gene expression caused by CuO nanoparticles can be explained by the cytoplasm and the nucleus’s Cu ion release, with the subsequent intracellular accumulation of Cu. For example, gene expression modulation in BEAS-2B cells by CuO nanoparticles appeared to be mainly oxidative stress associated with cell cycle arrest and is distinct from CuO nanoparticles (Strauch et al., 2017).

### 3.6 Antibacterial mechanisms of MNPs

Antibacterial properties of nanomaterials to inhibit infection and facilitate wound healing, antibiotic delivery systems, detection of bacteria, and antibacterial vaccines to prevent bacterial infections are all examples of MNP applications. The antibacterial mechanisms of MNPs are insufficiently understood, although the generation of oxidative stress, metal ion release, and non-oxidative mechanisms are widely accepted. Antibacterial resistance would require simultaneous mutations of genes in the bacterial cell, and because of the numerous methods of action against bacterial cells, it is difficult to become resistant to MNPs (Dhull et al., 2019; Sánchez-López et al., 2020). In addition to gene manipulation, bacterial resistance could emerge via genetic material exchange, including transformation (the transferring of exogenous DNA into the host) and transduction (the transferring of exogenous DNA by a viral vector). Antibiotic resistance can be caused by several mechanisms, including enzymes such as lactamases and acetyltransferases (Lorenz and Wackernagel, 1994; Travisano, 2001; Sayavedra-Soto and Stein, 2011). Changes in permeability of membranes that limit antimicrobial agent penetration are also considered prevalent resistance mechanisms, for example, alternation in penicillin-binding proteins (PBPs) (Macheboeuf et al., 2006; Miyachiro et al., 2019). The kanamycin-ZnONPs were produced by cell-free extract of mint (*Mentha piperita* L.) leaves, and the minimum inhibitory concentrations (MICs) showed robust antibacterial activity against the tested pathogens (Saleha et al., 2023).

#### 3.6.1 Generating reactive oxygen species (ROS)

The generation of ROS by MNPs is a significant antibacterial mechanism. Redox-potential molecules, also known as ROS, are extremely reactive. Different MNP types reduce the amount of oxygen molecules in the air, resulting in different forms of ROS. The superoxide radical, hydroxyl radical, hydrogen peroxide, and singlet oxygen are the four main forms of ROS, and they vary in their dynamic and activity levels (Yu et al., 2020). Magnesium oxide (MgO) and calcium oxide (CaO) nanoparticles can produce hydrogen peroxide (H_2_O_2_) and hydroxyl (OH), while zinc oxide (ZnO) nanoparticles do not produce any of them. However, metallic nanoparticles composed of copper oxide can produce all the above-mentioned types of radical molecules (Choudhury et al., 2017; Xue et al., 2018; Peng et al., 2020). Based on research studies, hydrogen peroxide (H_2_O_2_) generates fewer acute stress responses and can easily be balanced by using antioxidants, including catalase and superoxide enzymes, while hydroxyl (OH) and singlet oxygen (O2) induce microbial mortality. Under normal conditions, the bacterial cells can balance the generation and clearance of ROS. Conversely, when the cell produces too many reactive species, the redox potential strongly favors oxidation. This imbalanced condition creates oxidative stress, which harmfully affects the particular bacterial cells’ components. Studies have confirmed that excessive oxidative stress plays a major role in altering membrane permeability, which consequently leads to bacterial cell death. (Zhao and Drlica, 2014; Li et al., 2021). Previous studies have confirmed that aluminum oxide (Al_2_O_3_) nanoparticles, after penetrating the bacterial cell membrane, change the membrane permeability by generating oxidative stress inside the cell (Mestres et al., 2016). Similarly, AgNP ions facilitate catalytic activity and generate reactive hydroxyl radicals (OH) and reactive free radical oxygen ions ( $O_{2}$ ) that inhibit or kill bacteria by reaction with biological substances such as DNA (Gracien et al., 2019).

Many research outcomes have also demonstrated that ROS play an important role in DNA and bacterial cell interactions. ROS play a crucial role in upgrading the gene expression level of some proteins with oxidative potential, which is a vital step in the apoptosis of bacterial cells. The production of ROS disturbs the active components, which are responsible for preserving the regular physiological functions and morphological structure of the microorganism (Yu et al., 2020). For example, TiO_2_ nanoparticles induce electron-hole pairs (EHP) after light absorption. On the surface of the nanoparticles, extremely chemically active ROS are generated from the reaction of electron-hole pairs with water and air, which can attack intracellular bacteria and organic materials (You et al., 2016; Hu, Li et al., 2019). For example, zinc can be activated under visible and ultraviolet (UV) light, leading to extremely reactive ROS. Due to their negatively charged nature, hydroxyl and superoxide radicals can remain on the surfaces of cells and cannot penetrate the bacteria, while hydrogen peroxide (H_2_O_2_) can penetrate cells (Khokhra et al., 2017). Ultrasonic stimulation can potentially cause the creation of ROS. Consequently, ROS penetrate the cell and kill bacteria. Furthermore, metallic ions are quickly released under ultrasonic conditions to inhibit bacterial multiplication, which might be related to increased intracellular oxygen levels, nutrition, and metabolic waste movement caused by ultrasound (Huang et al., 2017; Kamineni and Huang, 2019).

#### 3.6.2 Non-oxidative mechanisms

To investigate the antibacterial processes of MgO nanoparticles, researchers have used electron paramagnetic resonance, proteomics techniques, Fourier transform infrared (FTIR), liquid chromatography-mass spectrometry (LC-MS), transmission electron microscopy (TEM), and flat culture techniques. In addition, MgO nanoparticles have been shown to have suitable antibacterial effects in different light conditions, including natural light, UV light, and darkness. The antibacterial effects of MNPs are not dependent on the oxidation of membrane lipids, which is caused by oxidative stress (Nguyen et al., 2018; Bhattacharya et al., 2021). Even when the bacterial cell membrane ruptures and the pores are evident, the initial MgO nanoparticles are not seen in the cell. Furthermore, some MgO nanoparticles can detect quantities of ROS. Administration of MgO nanoparticles had no influence on the levels of phosphatidylethanolamine (PE) and lipopolysaccharide (LPS) in the cell membrane, indicating that MgO nanoparticles do not promote lipid peroxidation. The amount of ROS-related protein in the bacterial cell did not increase, but many crucial metabolisms associated with proteins, such as the metabolism of amino acids, metabolism of carbohydrates, energy, and metabolism of nucleotides, were significantly decreased (Leung et al., 2014; Tang and Lv, 2014; Wang et al., 2017).

## 4 Metal-based nanoplatforms from bench to bedside

One limitation of efficient treatment is that it depends on good heterogeneity in tumor sites and subpopulations. Presently, the homogeneity of size distribution is not acceptable for cancer treatment. Despite the optimistic outcomes for nanobiotechnology during drug delivery development, most candidates were unsuccessful in emerging into clinical translation. There is an important role for the clinically appropriate theranostic platform in the diagnosis and treatment of early-stage cancer (Chen et al., 2017; Kolenc Peitl et al., 2019). Multifunctional nanoplatforms have significant advantages over plain nanoparticles, including real-time monitoring of drug release, accumulation, and biodistribution at the targeted tissue and improved therapeutic efficacy. In addition, theranostic nanoplatforms might be part of treatment strategies, as well as the expectation of therapeutic outcomes, monitoring, and seeking personalized medicine. Another function of theranostic nanoplatforms is bioimaging, for instance, in a monitoring setting or optical guiding while cancer surgery (breast and melanoma) is ongoing, as well as potentially improving accuracy (Blau et al., 2018).

At the University Cancer & Blood Center (UCBC), LUTATHERA^®^ and Xofigo^®^ are used to treat adults with GEP-NETs and prostate cancer, respectively. The FDA has authorized the trial of new PSMA theranostics in metastatic castration-resistant prostate cancer (mCRPC) (Malcolm et al., 2019). The CriPec^®^ platform enables the rational design of special nanomedicine with suitable therapeutic profiles. The tunable polymers and drug (Docetaxel) linkers in the CriPec^®^ nanoplatform are conjoined with therapeutics to develop customized nanomedicine. A new ligand can be added to the nanoplatform to tune it if selective tissue targeting is needed.

A GuIX^®^ nanoplatform has been made of gadolinium chelates and polysiloxane and has recently been accepted in phase I clinical trials. This theranostic platform has some advantages, such as no toxicity on two animal species, passive uptake in tumoral tissues owing to the EPR effect, and renal elimination after administration (Lux et al., 2019). A report of the first experience of pancreatic ductal adenocarcinoma (PDAC) patients treated with NBTXR3^®^ indicated that the primary feasibility of local NBTXR3 delivery could be activated by radiation, and it is suitable for patients who are not eligible for surgery (Bonvalot et al., 2019; Bagley et al., 2022). Those theranostic metal-based nanoplatforms are under investigation for phase I and II clinical trials or have FDA approval for different types of cancer, according to, as shown in Table 4.

**TABLE 4:** Some cancer theranostics in the clinical trial stage.

Cancer	Nanoplatform	Application	Therapeutic agent	More information/identifier
Glioblastoma	AuNPs	Therapeutics and diagnostics	NU-0129	Northwestern University, Collaborator: National Cancer Institute (NCI) Early Phase 1, Identifier: NCT03020017. (last update: 27 October 2020)
Head & neck	SPIONs	Therapeutics and diagnostics	Ferumoxytol	M.D. Anderson Cancer Center Early Phase 1, Identifier: NCT01895829. (last update: 21 September 2021)
	Silica-NPs	Therapeutics and diagnostics	Fluorescent cRGDY-PEG-Cy5.5-C dots	Memorial Sloan Kettering Cancer Center Phase 1 and Phase 2, Identifier: NCT02106598. (last update: 24 February 2022)
Gynecologic	Gd	Therapeutics and diagnostics	Polysiloxane Gd-chelate-based nanoparticles (AGuIX), cisplatin	Gustave Roussy, Cancer Campus, Grand Paris, National Cancer Institute, France Phase 1, Identifier: NCT03308604. (last update: 7 July 2021)
Lung	AuNPs	Therapeutics (AuroLase therapy) and diagnostics		Nanospectra Biosciences, Inc Not Applicable, Identifier: NCT01679470. (last update: 3 November 2016)

## 5 Challenges in clinical trial translation

Despite the preclinical advancements detailed above, metallic nanoparticle (MNP) therapeutic applications have faced many other important barriers to FDA approval that have yet to be overcome. Collaborations between research labs working on metallic nanoparticles and the FDA are needed to diminish the limitations and support the companies in carrying out clinical trials. The cost demanded to develop the formulations of metallic nanoparticle (MNP) platforms and the absence of an approved MNP precedent have discouraged researchers from following clinical translation, even though iron oxide nanoparticles (maghemite/magnetite) have been approved by the FDA for applications such as cancer diagnosis, hyperthermia therapy for cancer, and anemia (Soetaert et al., 2020; Materón et al., 2021). The new trend has made it especially challenging to justify the objective of therapy-based metallic nanoparticles (MNPs) over biodegradable nanoparticles such as polymers and vesicles in delivery systems.

Some prominent groups are focused on clinical trial translation and have moved to non-metallic nanoparticles when expanding translational therapies. Current evidence regarding the long-term biocompatibility of MNPs *in vivo*, combined with the continued lack of improvement in MNP clinical trials, has contributed to a lack of faith in MNP translational therapies. To date, Au-Si nanoshells have shown acceptable results in phase 1 clinical trials. However, some considerations remain for formulations of Au-based nanotherapeutics, and it is challenging to compare the outcomes of toxicity and distribution investigations of MNPs among various preparation methods, sizes, shapes, and surface charges. Furthermore, *in vitro* and *in vivo* analyses do not always correlate, which makes appropriate characterization for cytotoxicity expensive and time-consuming. Generally, the surface modification used to cover MNPs is believed to be degraded in blood circulation.

## 6 Conclusion and future perspective

Metallic nanoparticles (MNPs) have been used successfully in medical applications, including the delivery of drugs and genetic materials for the induction of cargo release. However, this research remains in the preclinical phases. The inadequacy of clear instructions for MNPs, minimal possibilities for supporting translational safety studies, and a few motivations for researchers to eliminate these limitations have resulted in voids in the clinical trials of MNPs. Furthermore, therapy evaluation, which leverages the valuable properties of MNPs, is a field of opportunity for improving clinically translational MNPs for target delivery and genetic manipulation. Nanotechnology has resulted in remarkable progress in synthetic approaches, which benefit the design and creation of different nanoplatforms (nanoparticles, nanotubes, nanocages, nanoshells, and nanodiamonds). MNPs and their biomedical applications in theranostics have advanced in recent decades, but various challenges have hampered their clinical translation.

MNPs can perform as extremely efficacious contrast agents in different biomedical imaging methods and provide many choices in cancer treatment. MNPs permit the delivery of multiple drugs to affect the target sites, which otherwise would not be achievable, and provide an essential basis for cancer treatment that demonstrates promising clinical trial consequences. It is predicted that continued discovery and investigation in nanobiotechnology will particularly affect future cancer treatment and biomedical imaging. In addition, the limitations of MNPs as drug vehicles, contrast agents in bioimaging, and sensitizers like toxicity must be investigated further, aiming to minimize the undesirable effects on the body.

Numerous challenges, including physicochemical properties, metabolism of drugs, the biocompatibility of MNP platforms, drug screening, surface properties, efficacy in both *in vivo* and *in vitro*, immunogenic issues, and cellular uptake, remain. Based on the ongoing challenges, achievable future directions include optimizing diverse MNPs and a full understanding of the exact mechanisms and interactions between the cell and MNPs to achieve adequate theranostic outcomes and accelerate the translation of MNPs into clinical trials.
